# Evaluating the technical efficiency of care among long-term care facilities in Xiamen, China: based on data envelopment analysis and Tobit model

**DOI:** 10.1186/s12889-019-7571-x

**Published:** 2019-09-05

**Authors:** Liangwen Zhang, Yanbing Zeng, Ya Fang

**Affiliations:** 1 State Key Laboratory of Molecular Vaccinology and Molecular Diagnostics, School of Public Health, Xiamen University, Xiang’an South Road, Xiamen, Fujian 361102 People’s Republic of China; 2 Key Laboratory of Health Technology Assessment of Fujian Province University, School of Public Health, Xiamen University, Xiang’an South Road, Xiamen, Fujian 361102 People’s Republic of China

**Keywords:** Long-term care, Technical efficiency, Quality, BCC, SBM, Tobit model

## Abstract

**Background:**

The technical efficiency (TE) of care among the elderly in long-term care facilities (LTCF) have become increasingly crucial policy concerns faced by developing countries and Asia, especially China. The purpose of this study was to evaluate the TE and the quality of care and identify its influencing factors among LTCF.

**Methods:**

A total of 32 registered LTCF in Xiamen of China were surveyed in 2016. The Banker-Charnes-Cooper (BCC) model and Slacks-Based Measure (SBM) model of Data Envelopment Analysis (DEA) were used to evaluate the TE of LTCF. The TE has been decomposed into pure technical efficiency and scale efficiency. Utilization of DEA with human, financial, and material resources as inputs and quantity, quality of nursing care as outputs allowed estimation of the relative TE of care in LTCF. In addition, this study applied SBM to measuring the efficiencies and slacks. Furthermore, Tobit model was performed to explore factors associated with TE.

**Results:**

There were 7 public and 25 private LTCF respectively, with a total of 6729 beds and 3154 elderly people. 17 LTCF were technically efficient (53.1%). In the BCC model, the average TE was 0.963. The average pure technical efficiency and scale efficiency of LTCF were 0.979, 0.984, respectively. There were 5 LTCF with increasing returns to scale, 8 LTCF with decreasing returns to scale. In the SBM model, the average TE was 0.813, and it had the same effective decision-making unit with SBM model. Depending on TE score from high to low, the top eight are private LTCF, and the last four were public LTCF. The slack analysis showed that they can be reduced in 8 LTCF with decreasing returns to scale such as 53.31% administrative staffs, 67.73% medical staffs, 33.1% caregivers, 51.66% paramedical staffs and 4.1% beds on average. The TE of private LTCF was higher than that of public LTCF. The LTCF in urban were more effective than rural. The TE of LTCF raised by increasing of working hours, training frequency and institutional occupancy.

**Conclusions:**

The overall TE of LTCF in Xiamen of China was relatively high, especially in private institutions. However, LTCF still needs to further improve the utilization of physical resources and the management and training of human resources. The TE of LTCF was associated to their location, institutional nature, allocation of human resources and occupancy rate. It was needed to focus on promoting the efficiency and quality of LTCF in order to achieve sustainability.

**Electronic supplementary material:**

The online version of this article (10.1186/s12889-019-7571-x) contains supplementary material, which is available to authorized users.

## Background

China is an upper middle-income country with the largest population of the elderly in the world. In 2016, China had 230 million elderly people aged 60 and over accounting for 16.7% of the total population, of whom 18.3% were elderly disabled and partially disabled. It was estimated that the number of adults aged over 60 will increase to 255 million (17.8%) by 2020, and that of adults aged 80 and over will reach 29 million [1, 2] in China. The accelerating aging of the population has brought about an increasingly vigorous demand for long-term care (LTC) for the elderly. Coupled with the changes in family structure, these have faced enormous challenges to the traditional family care. Living in long-term care facilities (LTCF) has become an important way to meet the needs of the elderly.

With the development of the national “welfare socialization”, the formulation and implementation of many old-age service policies and the influx of market funds have contributed to the rapid development of LTCF. However, these agencies have faced a series of problems such as inefficient services and poor care quality [3]. By the end of 2016, there were over 140,000 LTCF in China, an increase of 20.7% in the previous year [2]. Although the number of care beds for the elderly to grow rapidly, its utilization rate was not high. In 2014, the vacancy rate of China’s LTCF was as high as 48%. The occupancy rates of LTCF in both Shanghai and Beijing with a high degree of aging were less than 70%. The average occupancy rate of LTCF in Shandong Province was 46.74% and the lowest was less than 10% [4–6]. In addition, due to the late start of the construction of the pension service management system in our country, problems such as imperfect pension management system, weak service teams, and potential safety problems are highlighted. Unilaterally, expanding the scale of elderly care facilities even though we can meet the increasingly vigorous care needs in the short term, if we did not optimize the resource structure reasonably, it would inevitably affect the sustainable development of the LTC service system [6].

Efficiency can be simply used as a tool to explain the relationship between the inputs and outputs. And even technical efficiency (TE) can be applied to find out whether any waste can be eliminated without worsening any input or output [7]. For example, an organization was considered to be technically efficient if it produced the maximum desired outputs from the minimum inputs such as labor, capital, equipment, and technology. Data Envelopment Analysis (DEA) was defined as an advantageous non-parametric technique for evaluating performance in terms of relative efficiency in the presence of multiple inputs and outputs [8]. This method has been used to determine the best performer in various contexts.

Many studies have focused on the institutional planning of LTCF, including the exploration of institution building and service standards, service contents, and service personnel. However, there were few research on the efficiency measurement and resource allocation in China [5, 9, 10]. In the existing research on DEA method for efficiency evaluation, on the one hand, the selection of evaluation model was single, mainly focusing on the selection of the more traditional Charnes-Cooper-Rhodes (CCR) and Banker-Charnes-Cooper (BCC) models. The radial CCR model and BCC model suffer from one shortcoming; they neglected the slacks in the evaluation of efficiencies. To overcome this shortcoming efficiency scores can be computed using the “slack-based” non-radial and non-oriented DEA model (Slacks-Based Measure, SBM) [11]. However, existing literature lacked the research on the efficiency evaluation of SBM model. Relatively, there are few DEA studies which have evaluated TE by using the quality-of-care variables as outputs. In addition, a few studies have evaluated the TE that specifically focus on nursing care and nurse-related patient outcomes. Furthermore, only a few studies adopt Tobit model to analyze the influencing factors of TE value in the second stage of DEA [9].

In order to comprehensively improve the TE of LTCF and provide an evidence for decision-making through integrating and optimizing resource allocation so as to promote the transition of pension services to the sustainable development of increasing quality and improving efficiency, the purpose of this study was to evaluate the TE and quality using BCC and SBM models in the DEA method and identify its influencing factors among LTCF in Xiamen, China.

## Methods

### Setting and sample

A cross-sectional survey entitled “the Investigation on the Status Quo of efficiency and quality of care among the elderly in LTCF, Xiamen City” was performed in Xiamen, China, in 2016 (see Additional file 1). A total of 32 registered LTCF in Xiamen of China were surveyed from July 2016 to September 2016. The participants were mainly from the management, service personnel and the elderly of the LTCF. The survey covered the nature of the organization, operation mode, hardware facilities, occupancy rate, service contents, staffing, and caregivers. In order to ensure the orderly conduct of the investigation, standard operating procedures (SOPs) are formulated to provide uniform training for investigators. A questionnaire review will be carried out by those who are responsible for the recovery of the questionnaire, and the questionnaires that do not meet the requirements would be returned and amended.

### Statistical analysis

#### Selection of the model

Data Envelopment Analysis (DEA) was a non-parametric efficiency evaluation model, which was widely used in homogenous units because of its advantages in dealing with multi-input and multi-output indicators, such as the efficiency evaluation of medical services. DEA was used to estimate the relative technical efficiency of elderly care for nursing homes. The traditional DEA model mainly included CCR model based on constant returns to scale and BCC model with variable returns to scale [8]. Both models were radial models that rely on the basic assumption that inputs must be reduced as much as possible and outputs must be maximized as much as possible, but not in the real production process. The radial CCR model and BCC model suffer from one shortcoming; they neglect the slacks in the evaluation of efficiency. In addition, they neither could adequately account for the input-output redundancy problem. The calculated technical efficiency values were also relatively inaccurate and the decision-making units (DMU) could not be ranked. To overcome this shortcoming efficiency score could be computed using the “slack based” non-radial and non-oriented DEA model (SBM) [12].In addition, considering that the effective decision unit efficiency value was 1, Andersen et al. proposed a super-efficiency model (Super-efficiency DEA, SE-DEA) in 1993, allowing the effective decision-making unit to have an efficiency value greater than 1, so as to better sort. Tone (2002) combined the super efficiency model with the SBM model and proposed the super-efficient SBM model (SE-SBM) [13]. The SE-SBM model controlled the effects of slack variables, making the range of technical efficiency fluctuations small, and proved to be more reasonable measurement results in related studies (Liu Yaqian, 2011). In this study, BCC and SE-SBM models are both used to evaluate the technical efficiency of LTCF, quantitatively analyze the problems of under-input and excess efficiency, and rank the DMU. At the same time, the BCC model was used for efficiency evaluation and analysis, and compared with the results of the SBM model.

In this paper, BCC model and SBM model all choose input-oriented. The reason was that the output of LTCF was the care of the elderly, not controlled by the LTCF themselves. LTCF can only improve service efficiency by adjusting input. Therefore, we used the input-oriented model in this demonstration. An input-oriented DEA model was used to compute technical efficiency scores of nursing care can be expressed by the following formula.

$$ \left\{\begin{array}{l}\max\;\left[\theta -\varepsilon \left({e}^T\;{s}^{-}+{\hat{e}}^T\;{s}^{+}\right)\right]\\ {}\sum \limits_{j=1}^n{x}_j{\lambda}_j+{s}^{-}=\theta {x}_0\\ {}\sum \limits_{j=1}^n{y}_j\;{\lambda}_j-{s}^{+}={y}_0\\ {}{\lambda}_j\ge 0,j=1,\dots, n,{s}^{+}\ge 0;{s}^{-}\ge 0\end{array}\right. $$

In the case of *θ* = 1, *s*^−^ = 0, *s*^+^ = 0, the nursing home is fully efficient, whereas *θ* < 1 means that a nursing home is inefficient. BCC model adds constraint conditions on the basis of CCR model:

$$ \sum \limits_{j=1}^n{\lambda}_j=1 $$

At this time, it means that the return on scale of DMU remains unchanged and reaches the maximum output scale. In addition, when $\sum \limits_{j=1}^n{\lambda}_j=1<1$, this means that returns to scale are increasing. If the input of DMU is appropriately increased on the basis of the original input, the output will be increased by a higher proportion. Whereas $\sum \limits_{j=1}^n{\lambda}_j=1>1$, it means diminishing returns to scale, and increasing input does not lead to a higher proportion of output. In addition, for the formula of SBM-DEA, please refer to “SBM-DEA Model Based Efficiency Assessment of Public Sector Hospitals in Uttarakhand, India” for details [11].

Due to TE values have truncated characteristics and efficiency values are relative, using general multiple regression models results in bias and parameter estimation instability [14]. Based on the maximum likelihood function, the Tobit regression model estimates the parameters in an iterative manner. It was mainly suitable for analyzing dependent variables with truncated data and does not limit the distribution of independent variables. Therefore, this study applied it to the two-stage DEA model and analyzes the influencing factors of technical efficiency as the dependent variable by the BCC model and SBM model.

#### Indicators and variables

DEA measurement indicators included input and output variables. Considering the operability of decisions, input indicators should include items that decision makers have control over and can modify them [15]. As LTCF were labor-intensive industries. Scholars usually used fixed assets as capital input index and various types of institutional staff as human input index [16–19]. In terms of material inputs, the actual number of beds was a resource that was easily controlled by managers in material resources. A number of studies considered the number of beds as an indicator of material resources [5, 7, 20, 21]. In terms of output indicators, the quality and quantity of long-term care services serving as service agencies were the most important criteria for evaluation [17]. Therefore, we selected the fixed capital, service personnel and the number of beds to reflect the input capital, labor and material resources according to the literature selection method [22] and the accessibility of the index. The investment of fixed assets in our study included the cost of building, buying or renting houses, large equipment and so on. Currently, most LTCF pay more attention to data privacy, especially in financial aspects. Therefore, we can only collect their fixed asset input, which is a relatively representative standard formulated by government regulatory authorities. The output indicators covered the quantity and quality of services delivery. The number of elderly people with different care needs reflects their social benefits. The elderly was classified into three categories in this study: completely independent, partially disabled, and disabled, which measured by Katz Activities of Daily Living Scale, which included six basic activities of daily living: eating, toileting, bathing, dressing, getting in and out of bed and mobility. The quality of service is based on the fall rates of the elderly, the rate of complaint handling and the annual incidence of major accidents [5, 15, 19]. The final input indicators include six items: number of fixed assets, number of administrative staff, number of medical staff, number of caregivers, number of paramedical staff, and number of beds. And six output indicators: number of independent seniors, number of partially disabled seniors, and number of disabled seniors, fall rates, the rate of complaint unhandled and annual incidence of major accidents (choking, lost, pressure ulcers, scald, other injuries, these had been regulated by the Chinese quality evaluation standards of LTCF released in 2015).

This article included both environmental and management factors in the Tobit regression model, where the environmental factors include the geographical location [15, 19] and the nature of the organization [15, 16, 23, 24]. According to the interview, the number and quality of caregivers in the management factors are the major factors affecting the service efficiency and there are few relevant studies in China. Therefore, this article focuses on the selection of management factors, consisting of working hours of staff, annual average number of training and whether the college graduates of the caregivers; occupancy rates, included in the regression model [15, 16, 19, 22, 23]. DEA Solver pro 5.0 was used to conduct the DEA efficiency calculation and STATA MP 14.0 was used to perform the Tobit model analysis.

## Results

Of these 32 LTCF, 7 were public agencies, and 25 were private agencies. Among them, there were 6729 beds and 3154 elderly people (60% women). Twenty-two LTCF were in urban areas and 10 in rural areas. The establishment of LTCF was generally less than 10 years (62.5%). In the aspect of housing source, the houses of public LTCF mainly came from the government for free allocation and the private houses are mainly leased. The average occupancy rate of 32 LTCF was 46.9%. The occupancy rate of private LTCF was higher, and the occupancy rate in rural areas was generally below 50%. According to the interview, there were operating deficits in 70% of LTCF, of which 78% were private LTCF (Table 1).

**Table 1:** The basic situation of the 32 LTCF

Characteristics	Ownership		Location		Total N (%)
	Public	Private	Urban	Rural	
Type of disability^a^					
Disabled	1	8	8	1	9(28.1)
Partially disabled	2	9	8	3	11(34.4)
Independent	4	8	6	6	12(37.5)
Operating years					
<5	2	9	7	4	11(34.4)
5~	1	8	6	3	9(28.1)
10~	4	8	9	3	12(37.5)
Housing source					
Free allocation	6	2	5	3	8(25.0)
Lease	1	14	12	3	15(46.9)
Privately-owned	0	9	5	4	9(28.1)
Number of beds					
<100	2	7	8	1	9(28.1)
100~	2	8	5	5	10(31.3)
300~	3	10	9	4	13(40.6)
Occupancy rate (%)					
<50	2	5	2	5	7(21.9)
50~	3	6	5	4	9(28.1)
70~	1	6	6	1	7(21.9)
90~	1	8	9	0	9(28.1)

^a^Note:The main types of daily activity ability of the elderly residents in LTCF, including independent, partially disabled and disabled seniors, respectively

To evaluate the TE of the LTCF we had taken six inputs, namely number of fixed assets (Input 1), number of administrative staff (Input 2), number of medical staff (Input 3), number of caregivers (Input 4), number of paramedical staff (Input 5), number of beds (Input 6), and six outputs namely, number of independent seniors (Output 1), number of partially disabled seniors (Output 2), number of disabled seniors (Output 3), fall rates (Output 4), the rate of complaint unhandled (Output 5) and annual incidence of major accidents (Output 6). Input-output indicators of the observations were not missing or negative. There was not any significant linear relationship between input and output indicators. The thumb rules “the number of DMUs was expected to be larger than twice the sum of inputs and outputs” was applied for the selection of a number of LTCF, inputs, and outputs. (Table 2).

**Table 2:** Descriptive Statistics of Inputs and Outputs

Variable	M	SD	Minimum	Maximum
Inputs				
Input 1 (million yuan)	2.2	3.68	0.5	16
Input 2	5.5	5.96	1	31
Input 3	5.5	8.40	1	36
Input 4	24	22.32	10	115
Input 5	7.5	8.57	1	35
Input 6	80	96.42	24	300
Outputs				
Output 1	11.5	20.44	6	100
Output 2	28.5	22.56	2	85
Output 3	33.5	53.27	4	249
Output 4 (%)	14.65	5.21	0	39.8
Output 5 (%)	2.04	2.17	0	18
Output 6 (%)	0.85	0.26	0	4.71

Table 3 shows that 17 LTCF were technically efficient (53.1%). In the BCC model, the average TE was 0.963. The average PTE and SE of the LTCF were 0.979, 0.984 respectively. There were 5 agencies with increasing returns to scale (IRS), 8 LTCF with decreasing returns to scale (DRS) and 19 LTCF with constant returns to scale (CRS). In the SBM model, the average TE was 0.813, and two models had the same effective DMU. The top 8 with better technical efficiency were private LTCF. Four of the last eight, which had poor technical efficiency, were public LTCF.

**Table 3:** The TE evaluation of the 32 LTCF

DMU	BCC Model				SBM Model	
	TE	PTE	SE	RTS	TE	Order
1	1.000	1.000	1.000	CRS	1.000	8
2	0.910	0.930	0.979	DRS	0.437	30
3	1.000	1.000	1.000	CRS	1.000	11
4	1.000	1.000	1.000	CRS	1.000	3
5	0.913	1.000	0.913	DRS	0.714	21
6	1.000	1.000	1.000	CRS	1.000	15
7	1.000	1.000	1.000	CRS	1.000	4
8	1.000	1.000	1.000	CRS	1.000	13
9	1.000	1.000	1.000	CRS	1.000	7
10	1.000	1.000	1.000	CRS	1.000	2
11	1.000	1.000	1.000	CRS	1.000	16
12	0.634	0.669	0.946	DRS	0.363	32
13	0.985	0.986	0.999	DRS	0.757	20
14	1.000	1.000	1.000	CRS	1.000	5
15	1.000	1.000	1.000	CRS	1.000	14
16	0.958	1.000	0.958	IRS	0.577	25
17	1.000	1.000	1.000	CRS	1.000	12
18	0.993	0.998	0.996	IRS	0.679	22
19	0.990	0.991	0.999	IRS	0.886	18
20	0.948	0.958	0.990	IRS	0.670	23
21	0.912	0.919	0.992	CRS	0.472	29
22	0.883	1.000	0.883	DRS	0.570	26
23	0.922	1.000	0.922	DRS	0.812	19
24	1.000	1.000	1.000	CRS	1.000	10
25	0.958	1.000	0.958	DRS	0.567	27
26	1.000	1.000	1.000	CRS	1.000	1
27	0.968	0.999	0.969	IRS	0.485	28
28	1.000	1.000	1.000	CRS	1.000	17
29	0.873	0.887	0.985	DRS	0.422	31
30	0.980	0.980	1.000	CRS	0.607	24
31	1.000	1.000	1.000	CRS	1.000	9
32	1.000	1.000	1.000	CRS	1.000	6

Note:*PTE* Pure technical efficiency; SE: scale efficiency; TE = PTE*SE

Due to the manpower and material resources invested in pension services in the field of health care were controllable by institutional managers and the output of services was more controlled by demand, the DEA model in this paper selected input-oriented measurement methods. The input-oriented was to compare the use of resources under the same output. Using the SBM model to calculate projection values of non-TE effective LTCF in terms of input and output, we could calculate the number of adjustments (actual value-projection value) and adjustment proportion (adjustment amount/adjustment value* 100%) of input and output in 8 LTCF with decreasing returns to scale. In order to improve TE, the slack analysis results showed that on average 53.31% administrative staff, 67.73% medical staff, 33.1% caregivers, 51.66% paramedical staff and 4.1% beds can be reduced in 8 LTCF with DRS. These 8 agencies were needed to reduce 6 administrative staffs, 10 medical staffs, 14 caregivers, 10 paramedical staff and 11 beds on average (Table 4).

**Table 4:** Adjusted volume and proportion of input indicators in 8 LTCF with DRS

DMU	Input 2		Input 3		Input 4		Input 5		Input 6	
	NA^a^	%	NA	%	NA	%	NA	%	NA	%
2	7	87.50	8	80.00	3	18.75	21	95.45	0	0.00
5	0	4.34	20	81.79	4	13.49	12	43.36	0	0.00
12	25	81.20	19	81.84	66	75.99	18	51.60	73	27.90
13	2	47.29	3	37.37	7	36.74	0	0.00	0	0.00
22	5	45.71	13	74.37	14	37.97	5	57.14	0	0.00
23	1	10.12	12	67.88	7	15.79	0	0.00	0	0.00
25	5	62.83	2	38.06	13	44.92	4	70.75	0	0.00
29	7	87.45	8	80.51	3	21.11	21	94.98	15	4.90
Mean	6	53.31	10	67.73	14	33.10	10	51.66	11	4.10

Note: ^a^NA: the number of adjustments

Technical efficiency was measured by the BCC model and the SBM model, respectively, and Tobit model was performed to explore the influencing factors of TE scores (Table 5). TE of LTCF was linked to their location, institutional nature, allocation of human resources and occupancy rate. The TE of private LTCF was higher than that of public agencies. The LTCF in cities were more efficient than rural ones. The longer the working hours of caregivers, the more training, the higher the institutional occupancy, and the better the service efficiency of institutions.

**Table 5:** Tobit regression analysis of the influential factors on TE of LTCF

	BCC Model				SBM Model			
	Estimate	*P*	95%CI		Estimate	*P*	95%CI	
Environmental Factors								
Location	−0.035	0.238	−0.095	0.025	−0.258	0.044*	−0.508	−0.007
Institutional nature	0.068	0.020*	0.011	0.124	0.150	0.187	− 0.078	0.377
Management Factors								
Working time ≥ 2 years	0.193	0.028*	− 0.023	0.409	1.101	0.019*	0.193	2.008
Annual number of trainings	0.013	0.000***	0.006	0.019	0.043	0.002**	0.017	0.069
Regular college graduate	−0.227	0.002**	− 0.397	−0.057	− 0.763	0.034*	−1.463	− 0.063
Occupancy rate	0.229	0.039*	−0.047	0.505	1.102	0.035*	0.085	2.118
Pseudo R^2^	37.489				0.678			
Likelihood ratio	17.135				−6.518			

Note:***P<0.001; **P<0.01; *P<0.05

## Discussion

According to the classical CCR and BCC models, it was not possible to include unused and inefficient inputs corresponding to slack variables into the assessment, while the SBM model can solve the problem of input-output redundancy and sequencing. Although the SBM model has been widely used in environmental engineering and other fields, it was seldom applied to the efficient evaluation of the health service system in China [25–27].Mogha S et al. (2015) estimated the pure technical efficiency and scale efficiency of 36 public hospitals in Uttarakhand region based on SBM model, and evaluated the utilization of medical resources in public hospitals [28]. Currently, only Liu et al. (2011) in China compared the measurement results of different DEA models and concluded that the SBM model could effectively remove the influence of relaxation variables on the evaluation results [23]. In our study, BCC and SBM models were utilized to evaluate the TE of LTCF, and further analyzes the influencing factors by SBM-Tobit two-stage method, which provided an important reference for future researchers on choosing the efficient evaluation methods for LTCF, and also to analyze the relationship between resource investment and inappropriate scale relationship to provide the evidence. In addition, in order to compare the results of BCC model analysis with those of previous studies, this study incorporates both BCC model and BCC-Tobit model. It is found that when there is slack in the input index, the measurement of technical efficiency by BCC model is higher than that by SBM model.

In this paper, the analysis of LTCF efficiency showed that the average technical efficiency values of BCC model and SBM model were 0.96 and 0.81, respectively, and the technically efficient DMUs in both models were 17 (53.1%). Compared with the previous research [10, 21], the average TE of LTCF in Xiamen was higher overall, but there were still some problems such as under-utilized scale resources, low technical efficiency and service quality. For example, eight LTCF in the BCC model were in a state of DRS, accounting for 25% of the total. Continuing to increase manpower and material inputs, these LTCF will reduce management efficiency, and excessive possession of resources will affect the optimization of the overall resource allocation of pension services. Five LTCF were in a state of IRS, which is conducive to the enhancement of service efficiency by increasing investment in resources. Comprehensive analysis of institutional returns on the scale and technical efficiency of the two models, we found that inefficient LTCF generally underinvested in the issue of under-utilization. On the one hand, LTCF with DRS should avoid blind expansion and put the appropriate manpower, material resources and capital into the scale of development of matching institutions. Managers should pay more attention to improve the professional level of service personnel, optimize the organizational management mode and improve the management efficiency. It was needed to focus on promoting the efficiency and quality of LTCF in order to achieve sustainability [29]. On the other hand, LTCF with IRS should also pay equal attention to personnel management while expanding their scale so as to provide quality and efficient retirement services. In short, the development of total pension resources should be combined with resource management and optimal allocation.

This study showed that private LTCF had higher TE than public LTCF, which were consistent with previous studies [22, 30]. On the one hand, it may be due to differences in the management model of the organization. On the other hand, it may be linked to the external policy environment and the nature of the agency. Private-owned LTCF were mainly for-profit business that increases revenue by attracting seniors. They had stronger management capabilities, more flexible methods, and a higher ratio of service personnel to the elderly, with better service efficiency and quality. Public LTCF are non-profit nature, mainly relied on government funding, bear the basic social security functions, the rigid management model may be the main reason for inefficiency. However, our research showed that there was a widespread problem of losses and operational difficulties in private organizations [31]. Therefore, the government should formulate and implement subsidies and preferential policies for private-sector organizations and fully encourage private-sector organizations to play a major role in pension services.

Different from previous studies [16, 20], this study found that institutional TE in urban was higher than that rural area, reflecting that the utilization rate of manpower and material resources in urban LTCF was higher than rural areas. According to interviews, the service quality of LTCF in rural areas was generally low, making it hard to attract the elderly. The higher the occupancy rate of LTCF, the higher the efficiency of their services [6]. In this study, the majority of occupancy rates were below 50% in rural LTCF, which may be a significant factor in the inefficiency of their services. The low occupancy rate reflects the contradiction between the unbalanced supply of pension resources and the needs of the elderly. High-quality pension resources are concentrated in the cities, which may be the basic problem of the structural supply and demand imbalance of pension services [27]. Therefore, the government should focus on the development of pension in rural areas.

Our study found that the proportion of caretakers working more than two years had a positive effect on technical efficiency. With the number of working year’s increases, caregivers’ skill levels of services continue to enhance, which can result in more efficient service delivery. The annual number of training for caregivers is positively related to efficiency. More training is taken, the more the caregivers’ care ability and efficiency is higher. Therefore, improving the stability of service personnel and strengthening the training of care workers was the keys to comprehensively improving the service quality and efficiency of the LTCF [16, 18]. The percentage of caretakers graduating from formal LTCF had a negative effect on efficiency. Combined with interviews, these caretakers have higher requirements for salary, career advancement and social acceptance, and greater mobility of staff, which may have contributed to the inefficiency of existing services. However, in the long run, the knowledge-based and specialized training of pension nursing personnel is its main development direction.

This study breaks through the limitations of previous studies on the selection of DEA models and indicators and comprehensively adopted SBM and BCC models to evaluate the TE and quality among 32 LTCF in Xiamen, China. The index selects comprehensive consideration of the quantity and quality of output indicators of LTCF, and further use the Tobit regression model to identify the influencing factors of TE. However, there are several limitations associated with this study. First, the data in our study were a cross-sectional survey and this limited the interpretation of our results, making it hard to draw causal conclusions. Second, the participants were sampled from one city in China, which may be local characterized and in turn exist some bias for interpretation in countrywide. Third, DEA was recommended to have homogenous units, however, public and private LTCF were both included in our study. Although they were different in the source of funds, their input and output indicators were same in most cases. Fourth, since DEA was a data-oriented method, the efficiency scores obtained were able to be directly affected by the combination of inputs and outputs used [32]. The selection of input-output indicators will vary according to research subjects, data access and researchers’ decisions. In this study, TE was calculated by using six variables as inputs and six quality-of-care variables as outputs based on the research purpose and data availability. However, due to the current income and expenditure of LTCF and related quality evaluation index was difficult to obtain, we will follow up with further research to make up for this shortcoming.

## Conclusion

In conclusion, the average TE of LTCF in Xiamen was higher overall compared with the previous research in China, but there were still some problems such as resource waste, low technical efficiency and service quality. TE of LTCF was associated to their location, institutional nature, allocation of human resources and occupancy rate. The TE of private LTCF was higher than that of public agencies. Therefore, we should focus on the following three aspects in order to enhance the service efficiency and quality of LTCF. First, managers of LTCF should optimize the allocation of resources, rationalize the layout of capital, labor, and material resources and reduce the waste of resources. Second, we should pay more attention to enhance staff’ quality/qualifications by training to replace the existing one-sided way to increase the number of personnel. LTCF should increase the intensity of professional training of service personnel, and constantly improve caregiver’s service ability and professionalism. Third, a standardized system for assessing the service efficiency and quality of care facilities should be explored and established in LTCF. In addition, the government should encourage and assist the development of private LTCF and give priority to supporting rural areas.

## Additional file

Additional file 1: The questionnaire used in this study. (DOCX 15 kb)

## Data Availability

Please contact the corresponding author for data requests.

## Abbreviations

BCC: Banker-Charnes-Cooper
CCR: Charnes-Cooper-Rhodes
DEA: Data Envelopment Analysis
DMU: Decision-making units
DRS: Decreasing returns to scale
IRS: Increasing returns to scale
LTCF: Long-term care facilities
PTE: Pure technical efficiency
SBM: Slacks-Based Measure
SE: Scale efficiency
SOPs: Standard operating procedures
TE: Technical efficiency
