# Supplementary material for: Evaluating the technical efficiency of care among long-term care facilities in Xiamen, China: based on data envelopment analysis and Tobit model
Source: BMC Public Health. 2019 Sep 5;19:1230. doi: 10.1186/s12889-019-7571-x (PMC6729073; doi:10.1186/s12889-019-7571-x)
Supplement: Supplementary file 1 — The questionnaire used in this study. (DOCX 15 kb) [file 12889_2019_7571_MOESM1_ESM.docx]

**The questionnaire used in this study（Abbreviated Version）**

1. **Basic information**
2. **Name of institution**
3. **Nature of the institution (public or private)**
4. **Fixed assets**
5. **Operating years**
6. **Housing area**
7. **Housing source**
8. **Number of beds**
9. **Occupancy rate**
10. **Staffing**
11. **Number of personnel（medical staff、administrative staff、paramedical staff、caregivers etc.）**
12. **Personnel training**
13. **Education level of personnel**
14. **Financial part**
15. **Charging standards for different items**
16. **Income and expenditure**
17. **Elderly residents**
18. **Type of disability**
19. **Annual fall rates** (%)
20. **Annual rate of complaint unhandled** (%)
21. **Annual incidence of major accidents** (%)

***Note: if necessary, please contact the corresponding author for a complete version of the questionnaire.***
